# Supplementary material for: Detection of Brain-Derived Cell-Free DNA in Plasma
Source: Diagnostics (Basel). 2024 Nov 13;14(22):2541. doi: 10.3390/diagnostics14222541 (PMC11592591; doi:10.3390/diagnostics14222541)
Supplement: Supplementary file 1 [file diagnostics-14-02541-s001.zip › FigureSuppl_3.pdf]

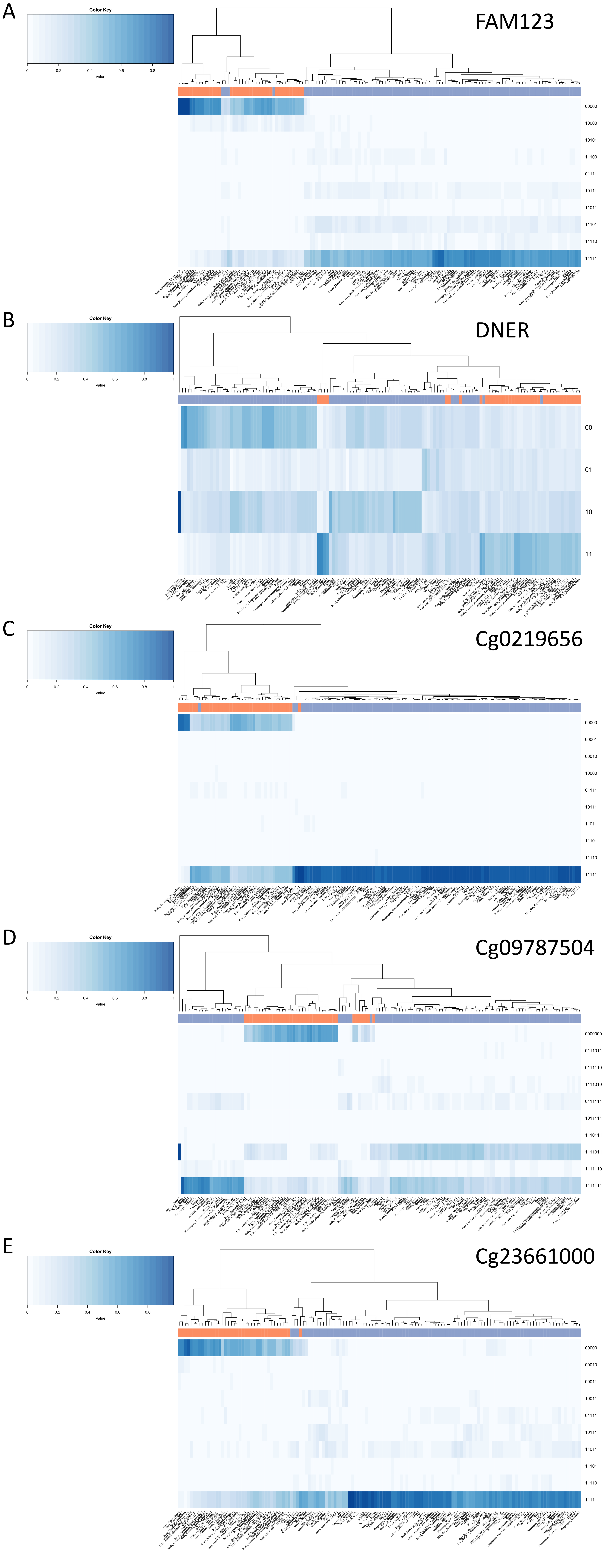
A
FAM123
Color Key
0 0.2 0.4 0.6 0.8
Value
00000
10000
10101
11100
01111
10111
11011
11101
11110
11111
B
DNER
Color Key
0 0.2 0.4 0.6 0.8 1
Value
00
01
10
11
C
Cg0219656
Color Key
0 0.2 0.4 0.6 0.8 1
Value
00000
00001
00010
10000
01111
10111
11011
11101
11110
11111
D
Cg09787504
Color Key
0 0.2 0.4 0.6 0.8 1
Value
0000000
0111011
0111110
1111010
0111111
1011111
1110111
1111011
1111110
1111111
E
Cg23661000
Color Key
0 0.2 0.4 0.6 0.8
Value
00000
00010
00011
10011
01111
10111
11011
11101
11110
11111
