# Supplementary figures and images for: Detection of Brain-Derived Cell-Free DNA in Plasma
Source: Diagnostics (Basel). 2024 Nov 13;14(22):2541. doi: 10.3390/diagnostics14222541 (PMC11592591; doi:10.3390/diagnostics14222541)

cg10094078 APC2

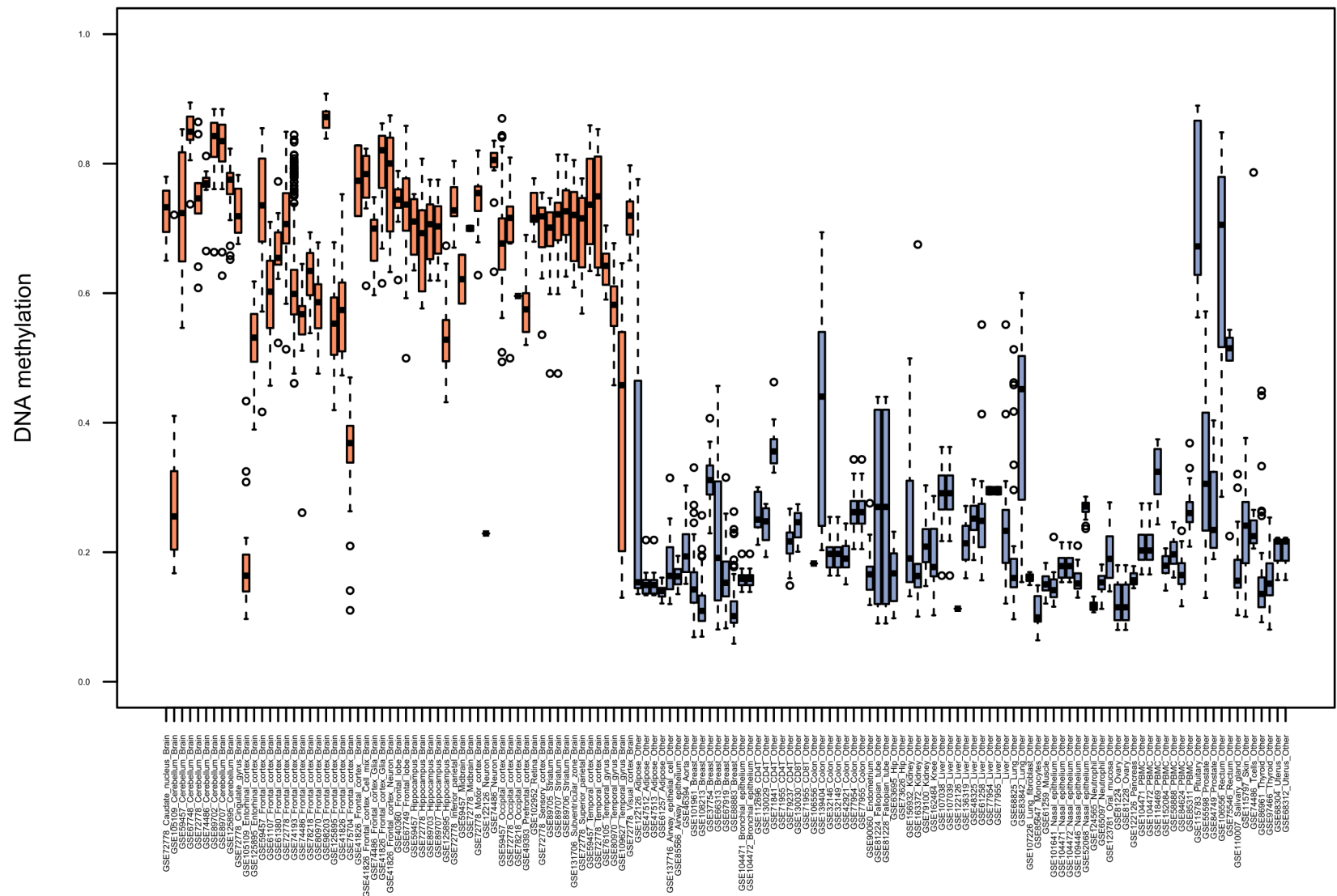

cg23661000 UBE4B

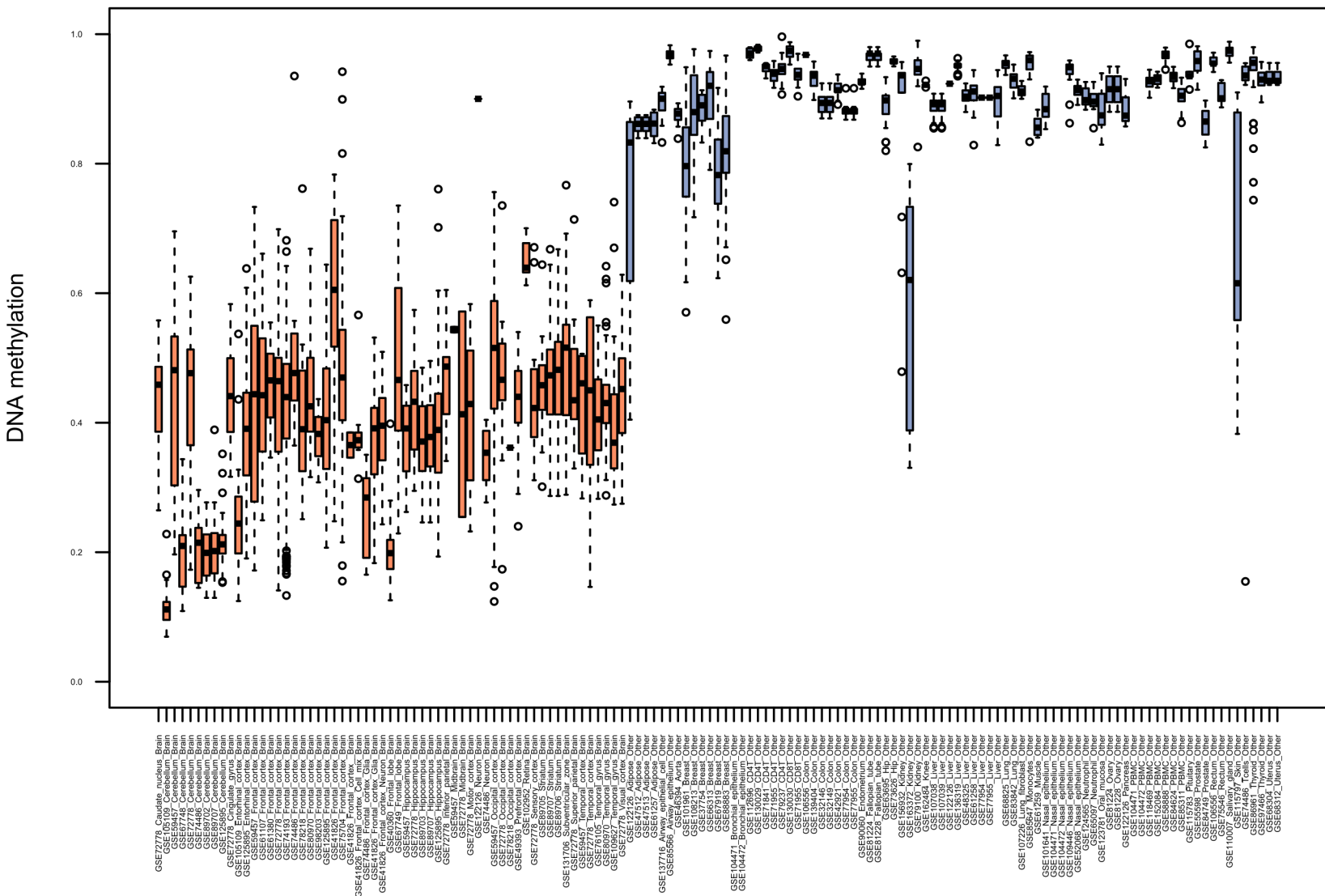

cg14115325 C10orf90

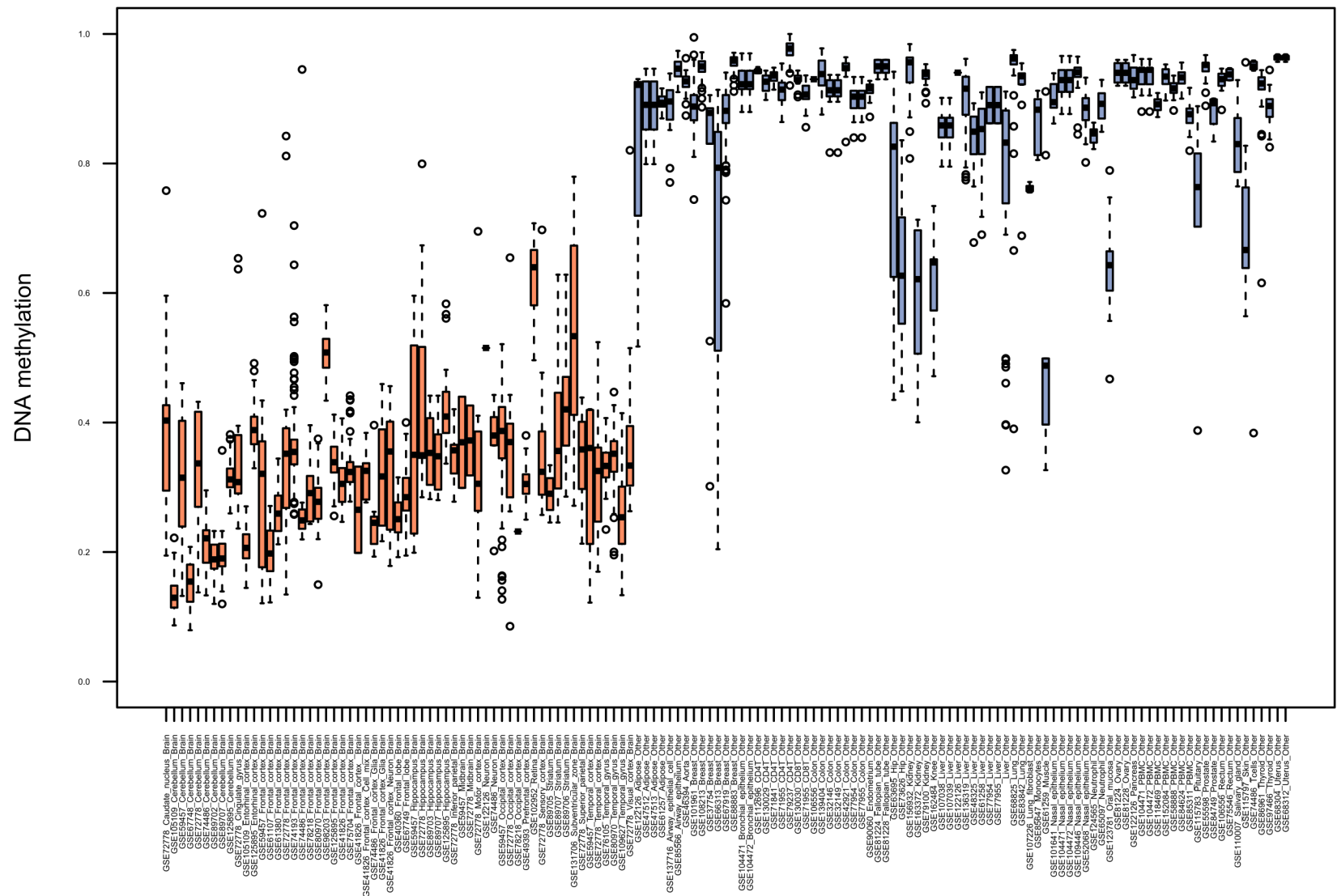

cg02619656 LINC01572

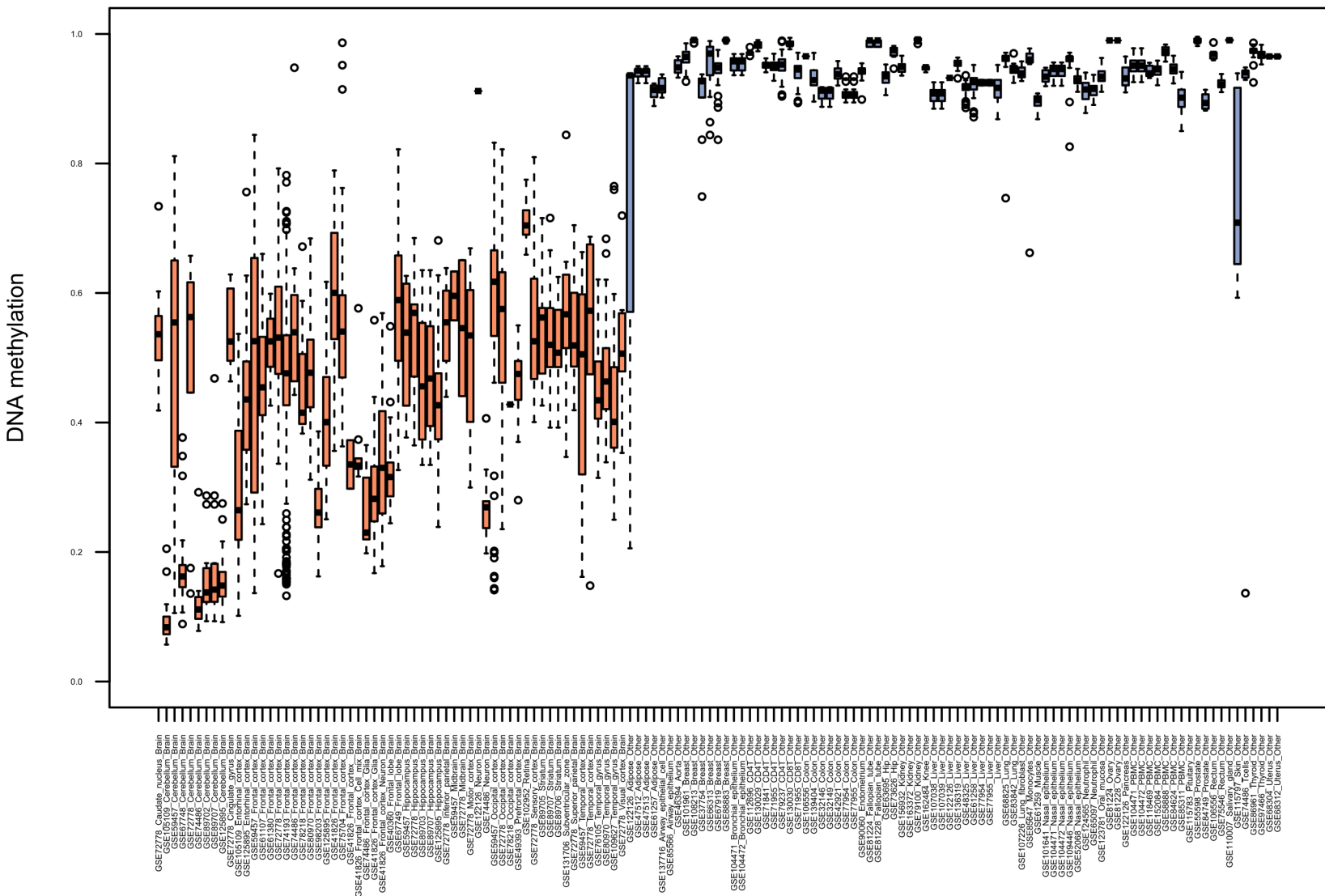

cg25541259 DNER

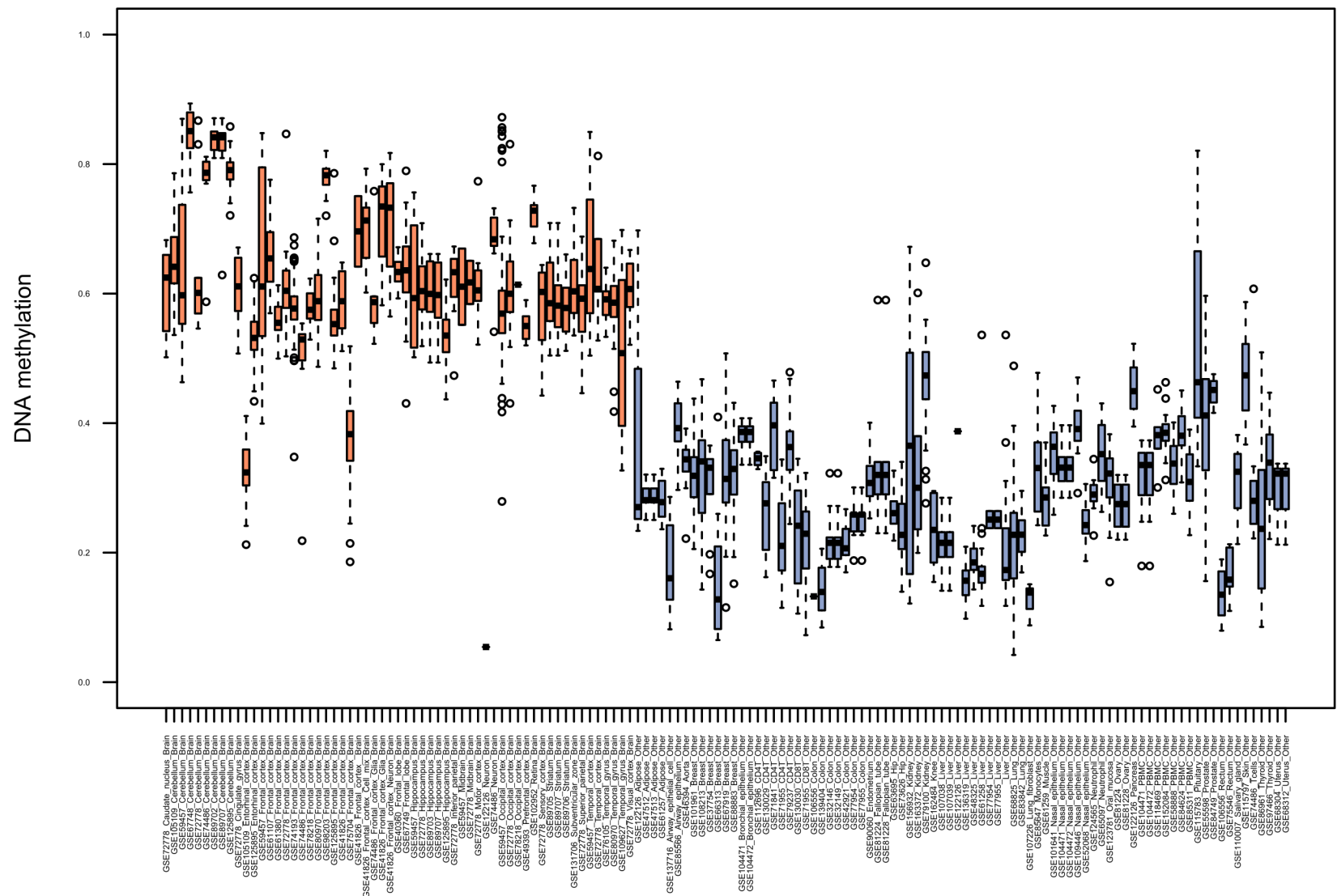

cg09787504 LINC02668

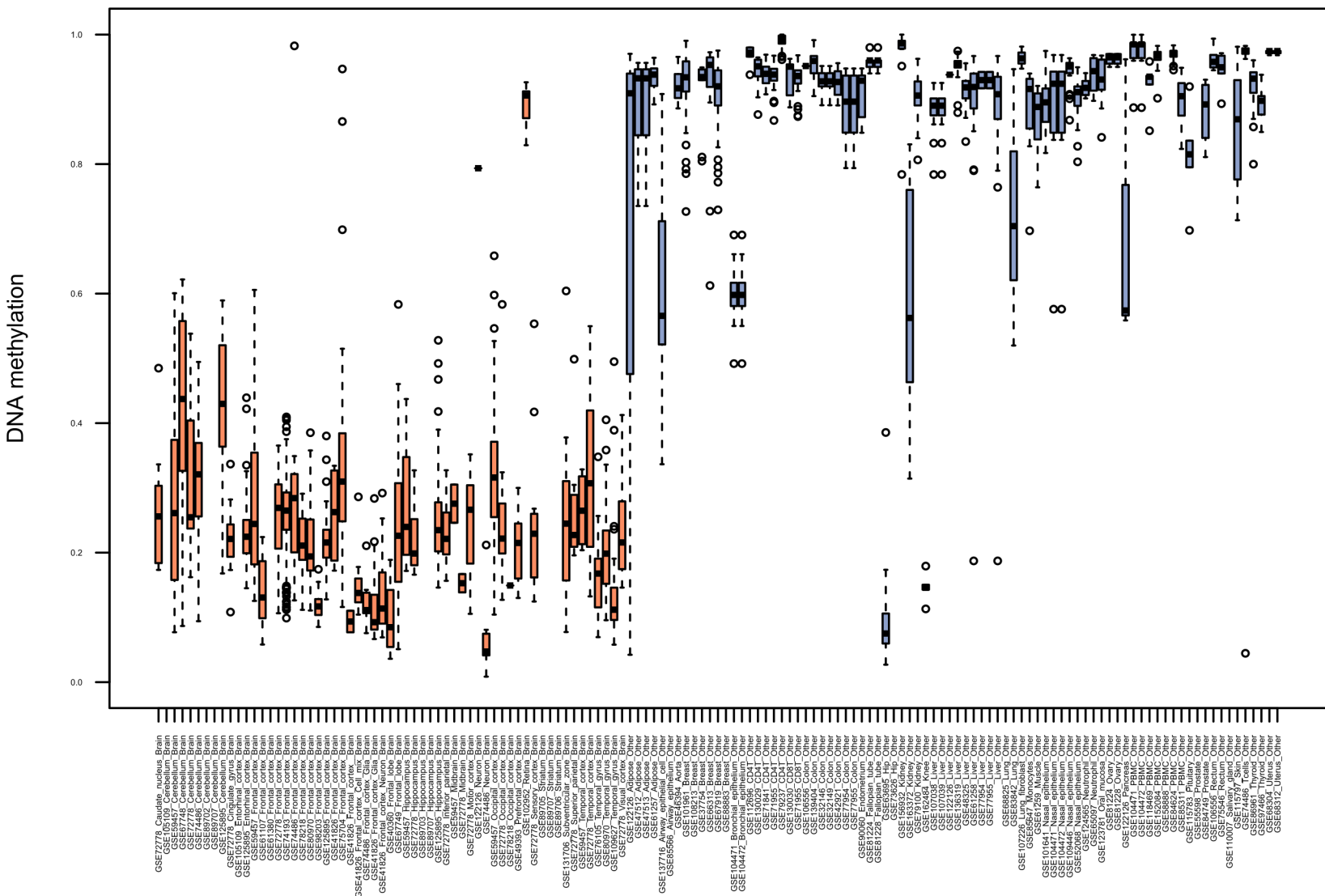

cg11724750 PACRG

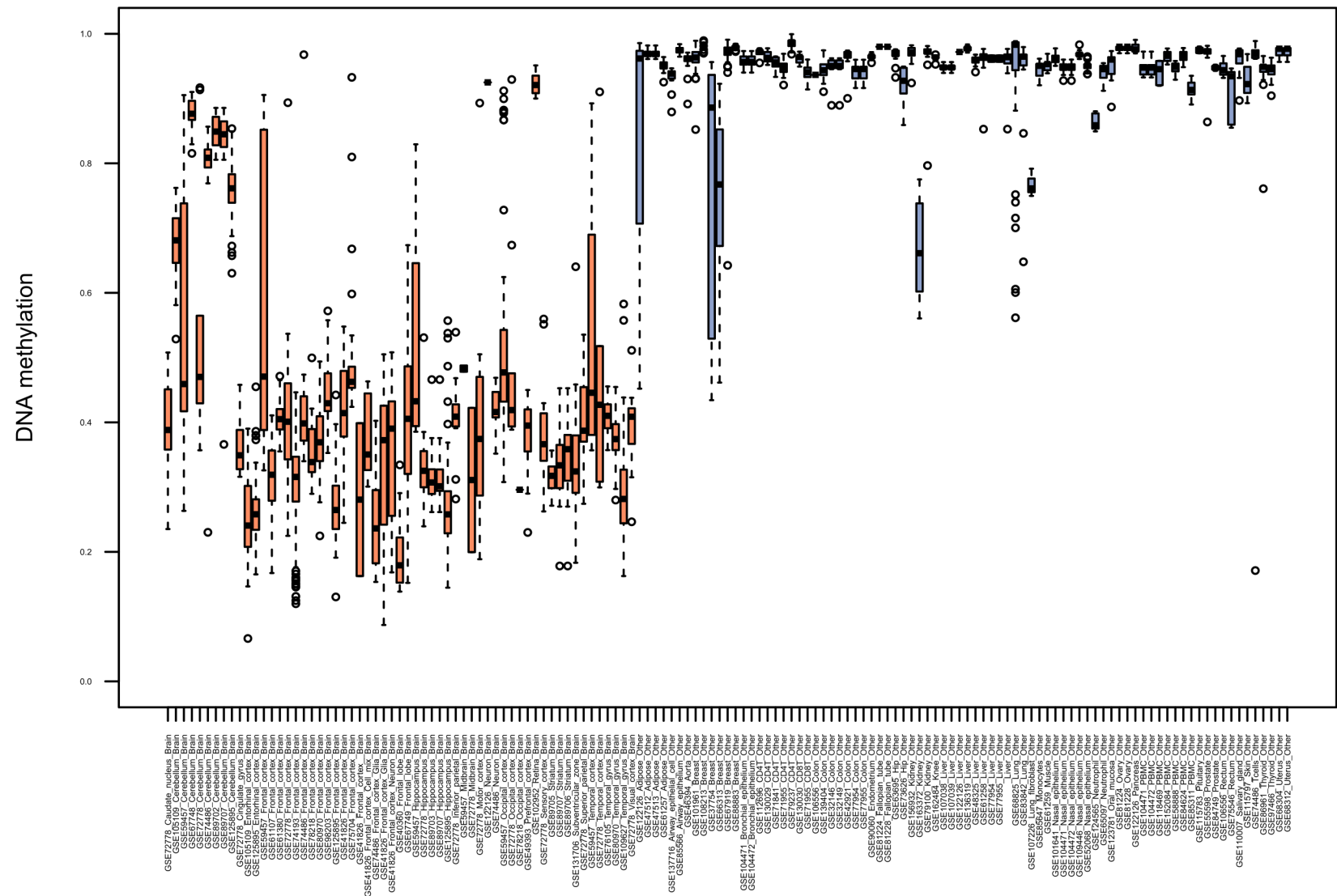

cg13055385 FAM123

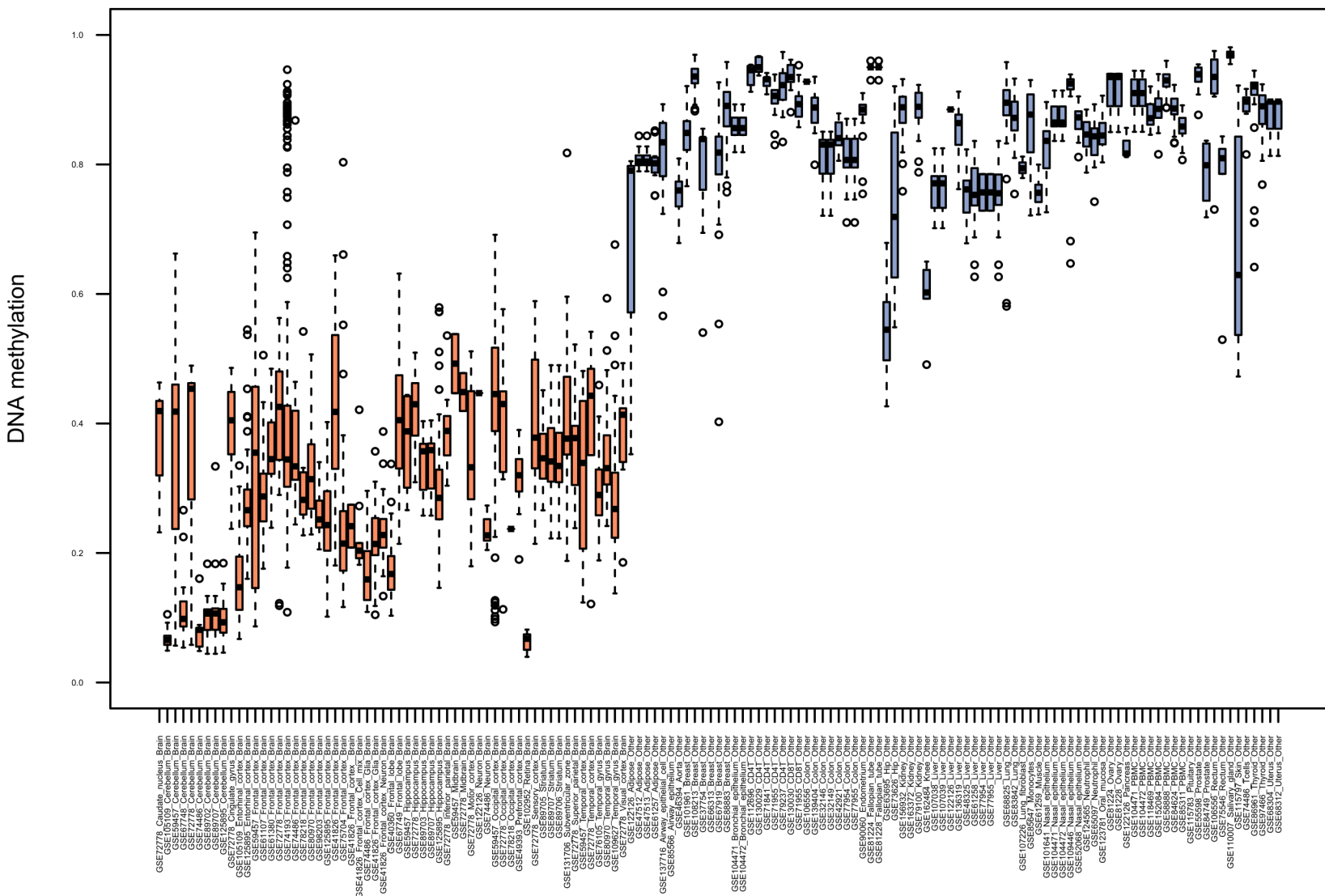

Supplement: Supplementary file 1 [file diagnostics-14-02541-s001.zip › FigureSuppl_1.pdf]

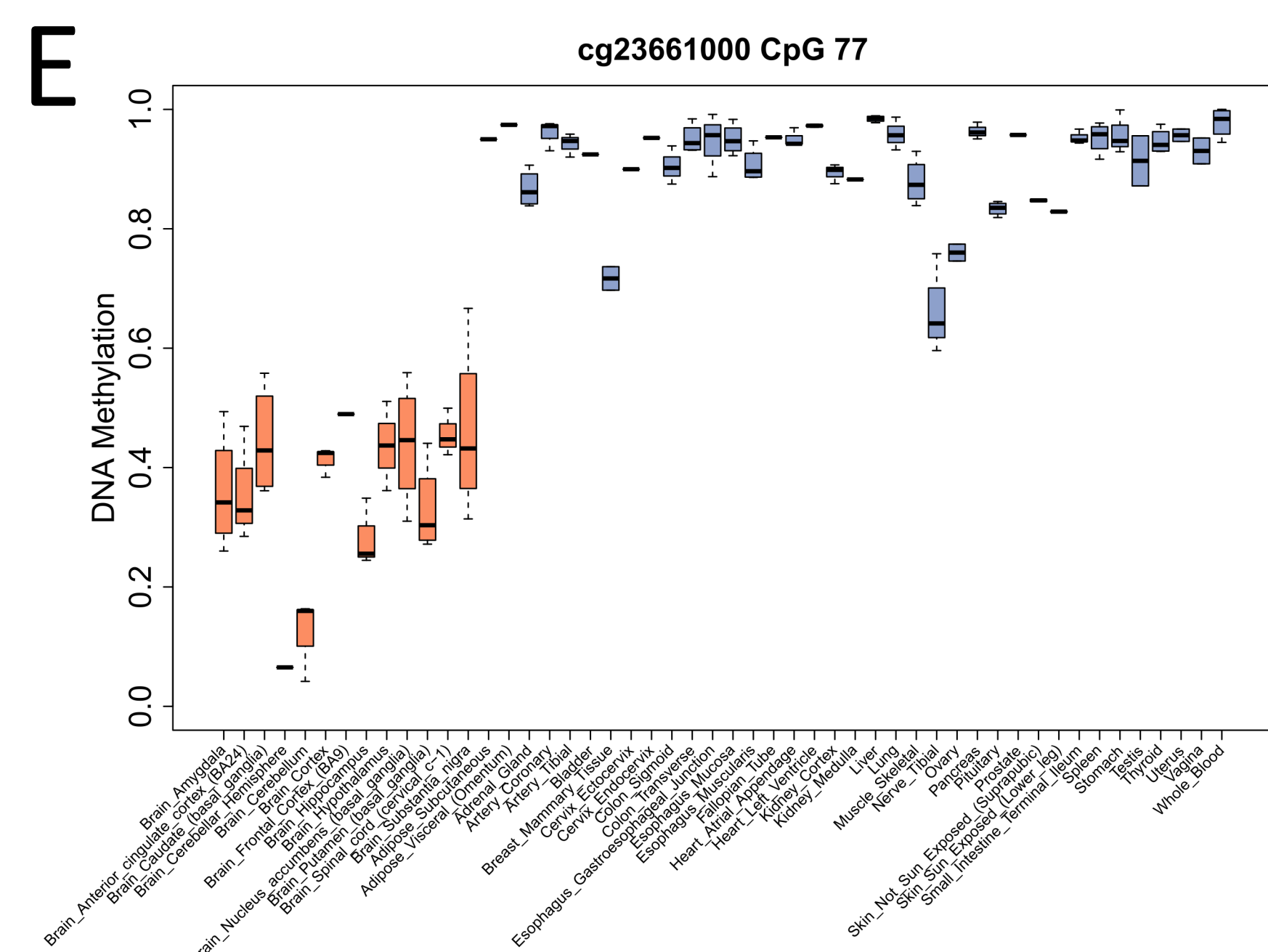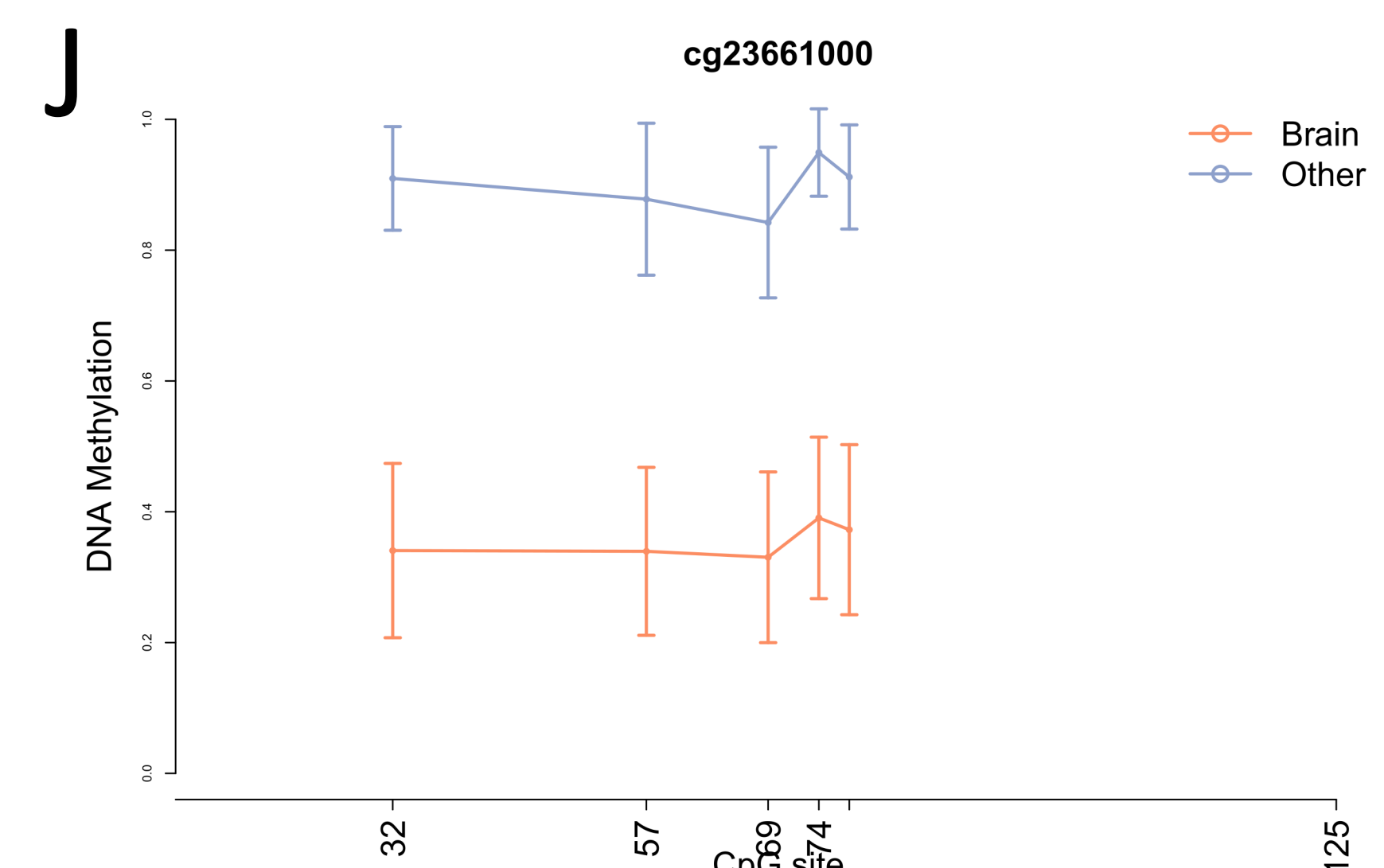

Supplement: Supplementary file 1 [file diagnostics-14-02541-s001.zip › FigureSuppl_2.pdf]

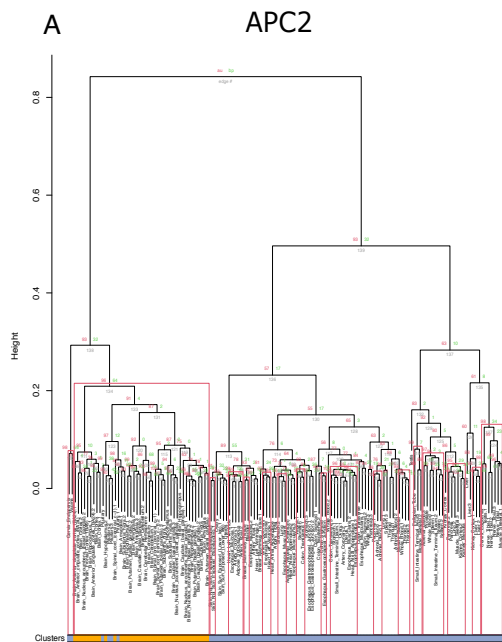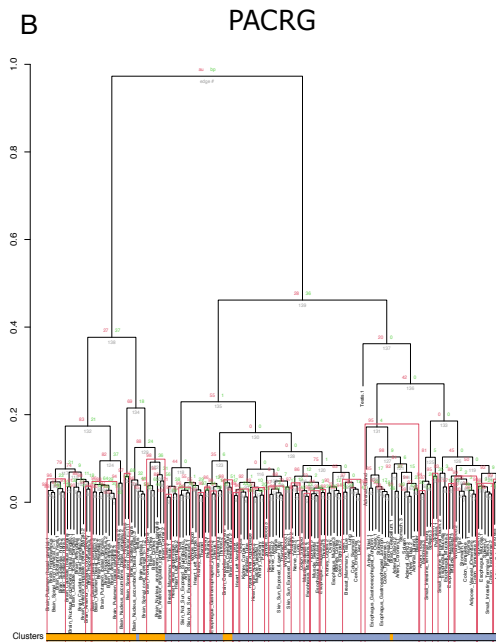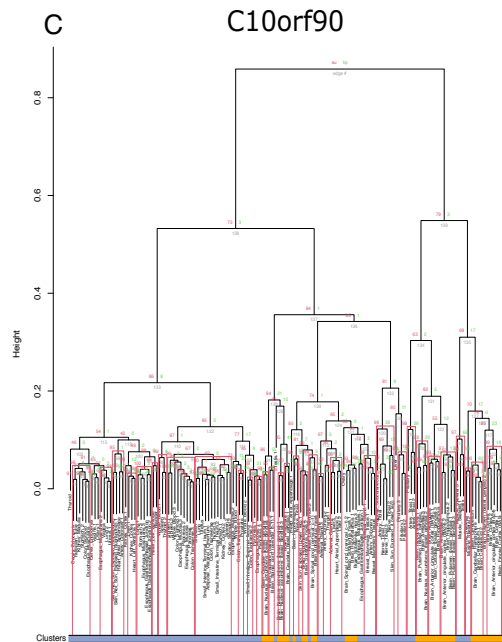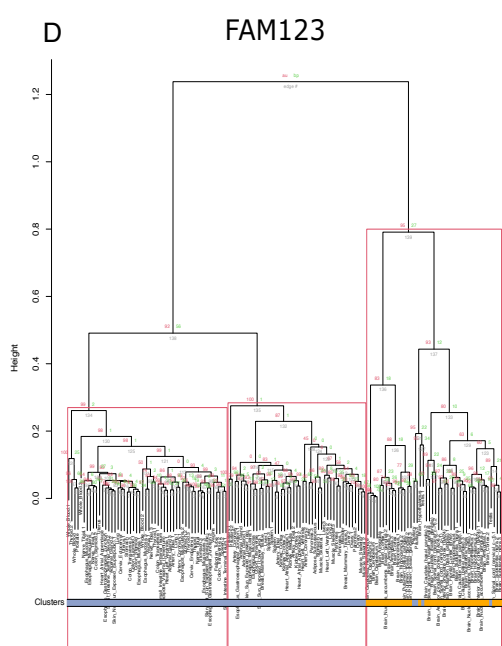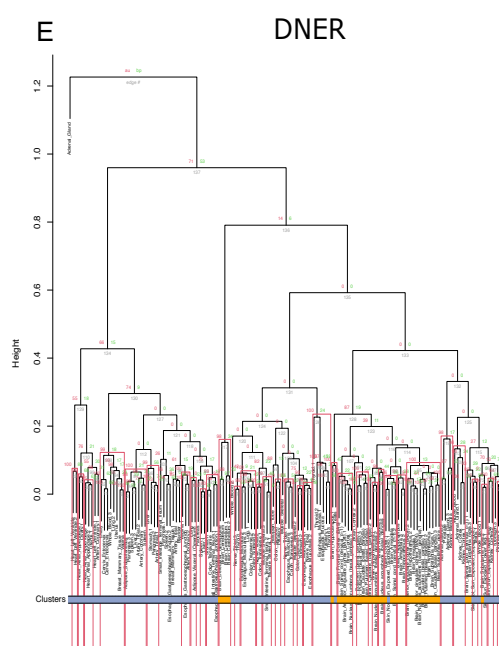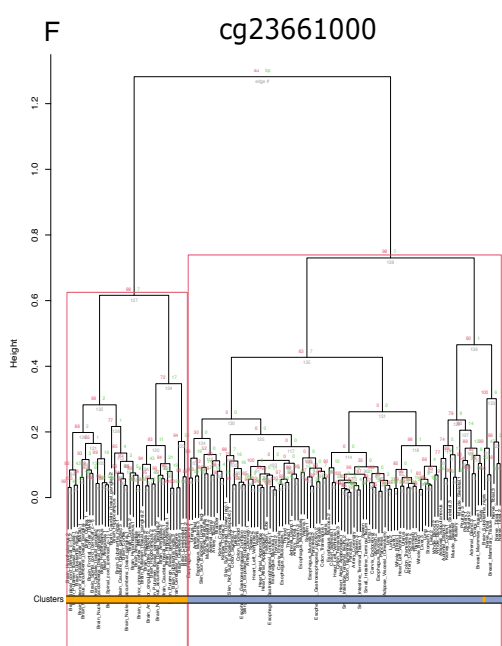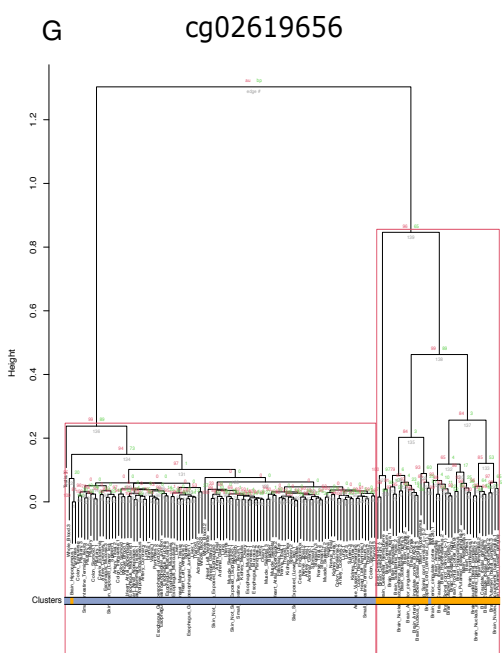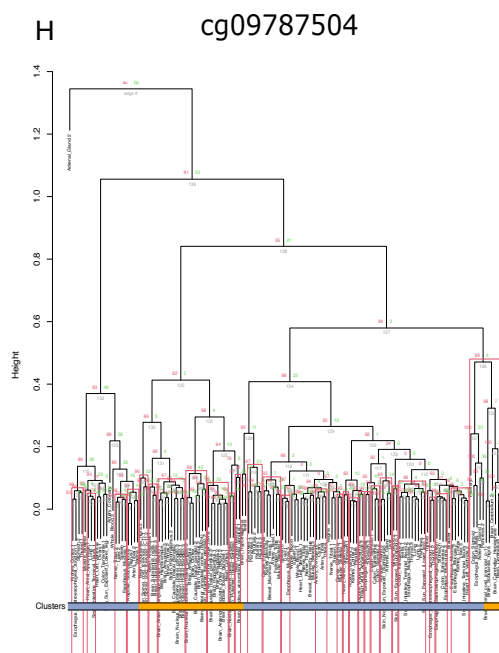

Brain  
Other tissues

Supplement: Supplementary file 1 [file diagnostics-14-02541-s001.zip › Supplementary Figure 4.pdf]

A

APC2

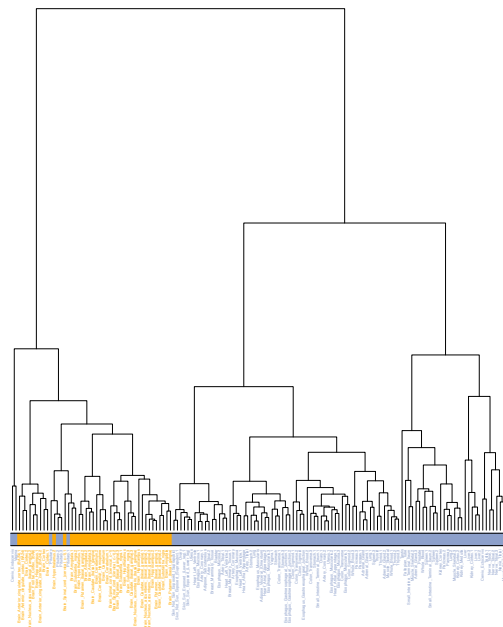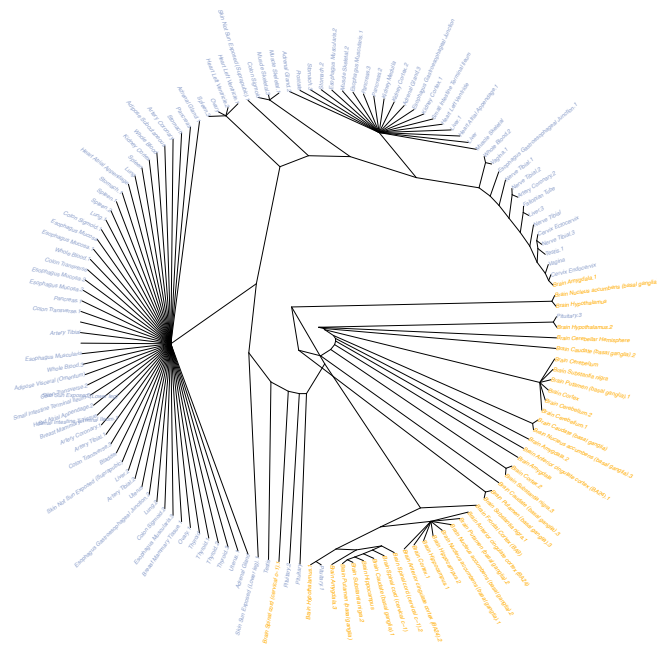

B

PACRG

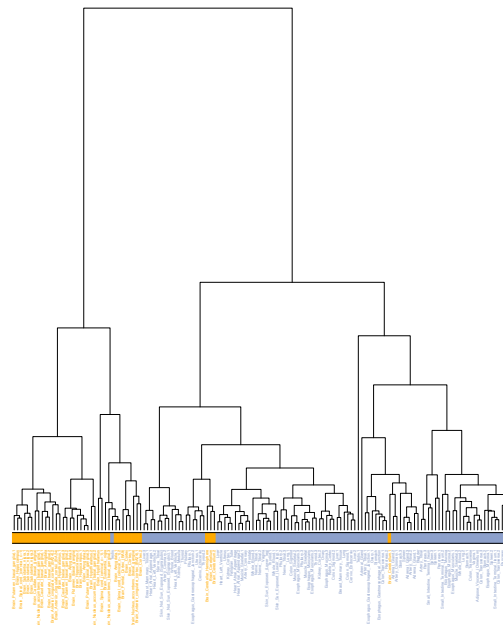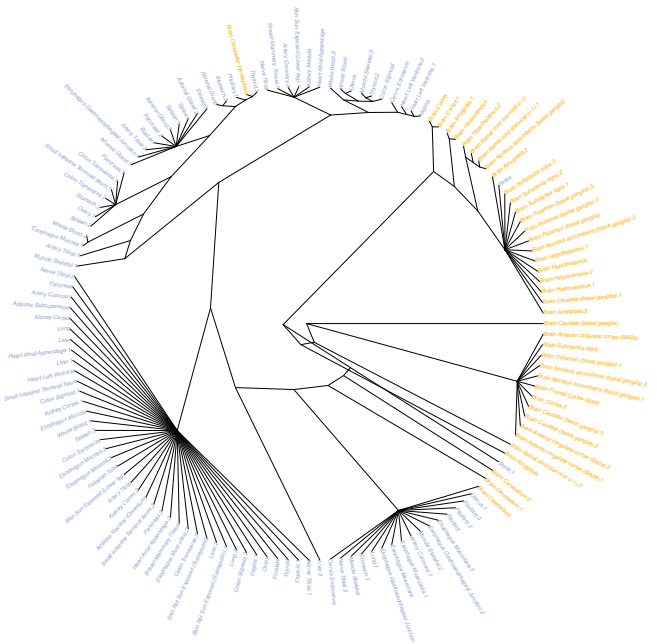

C

C100rf90

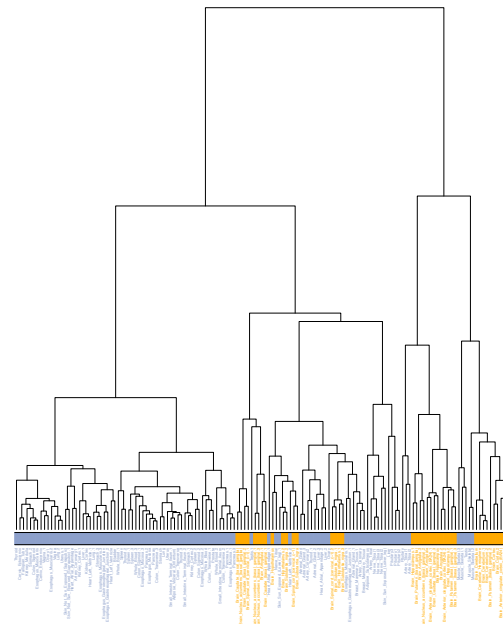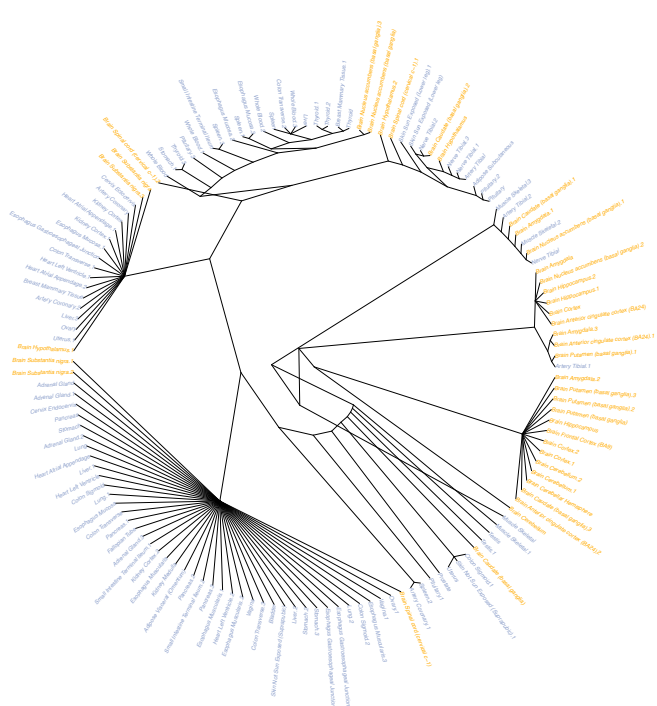

D

cg23661000

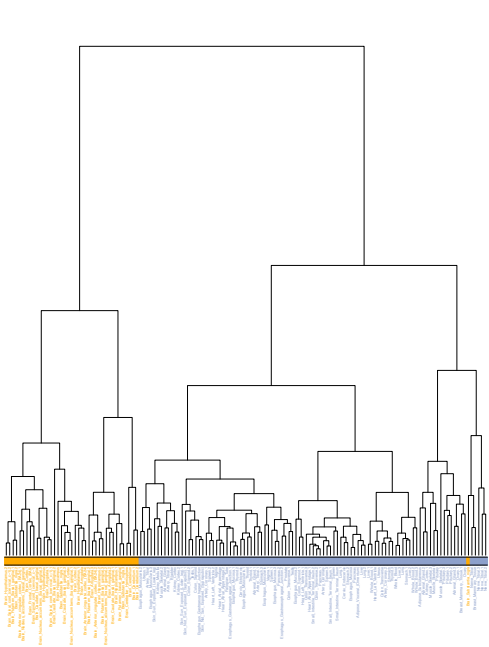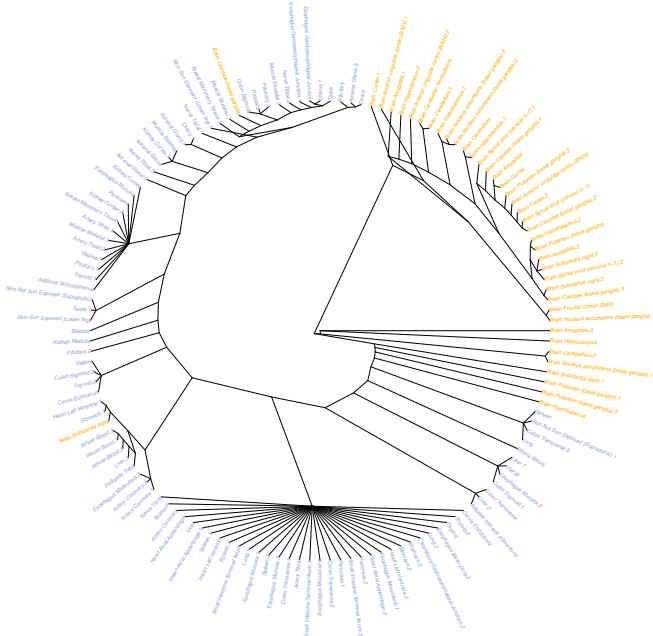

E

cg02619656

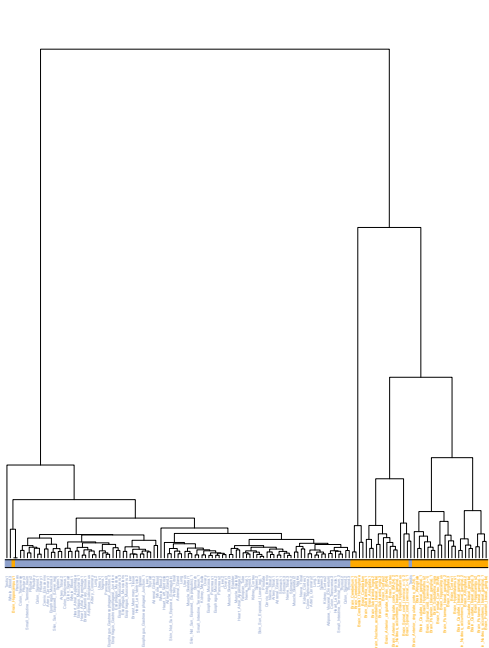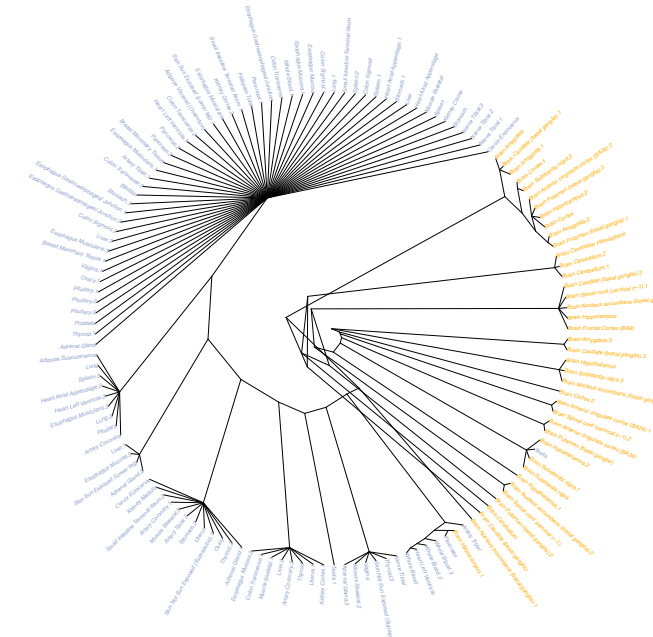

F

cg09787504

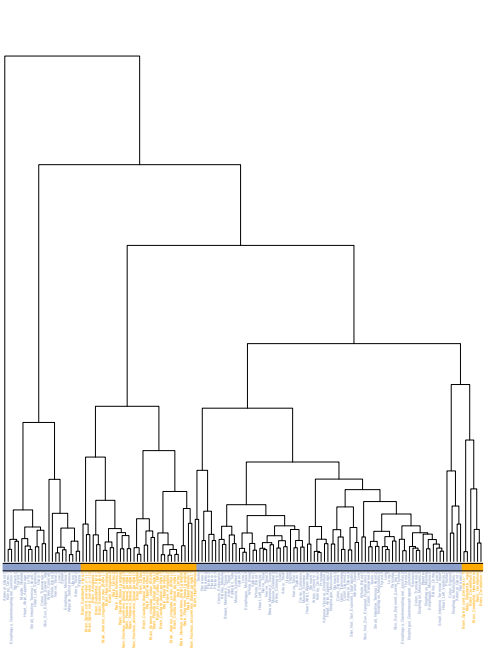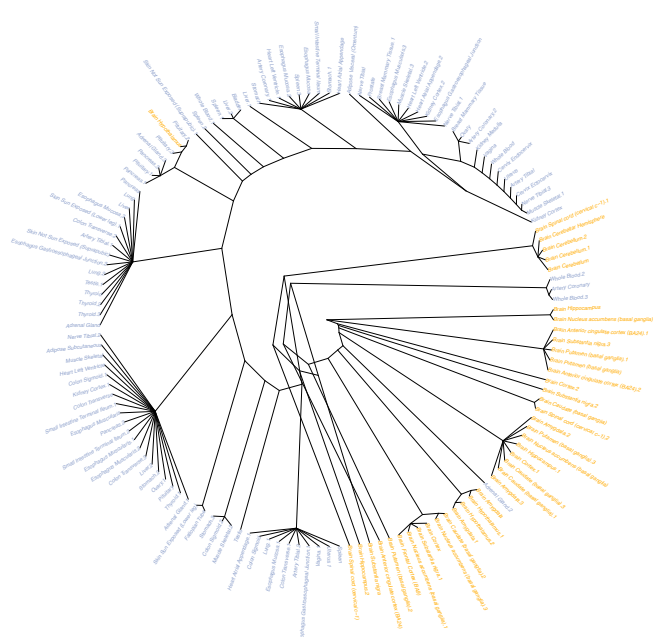

H

FAM123

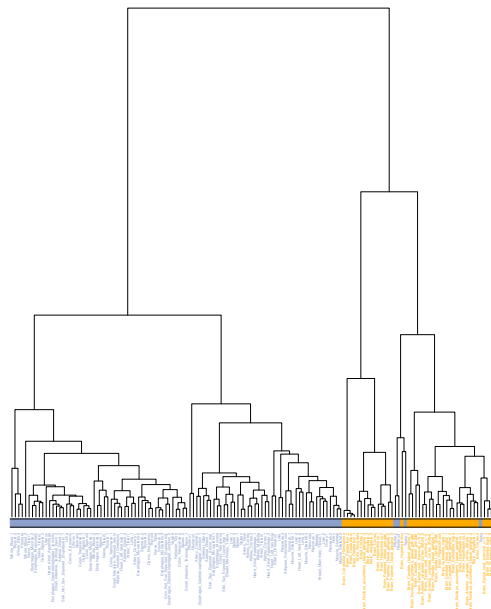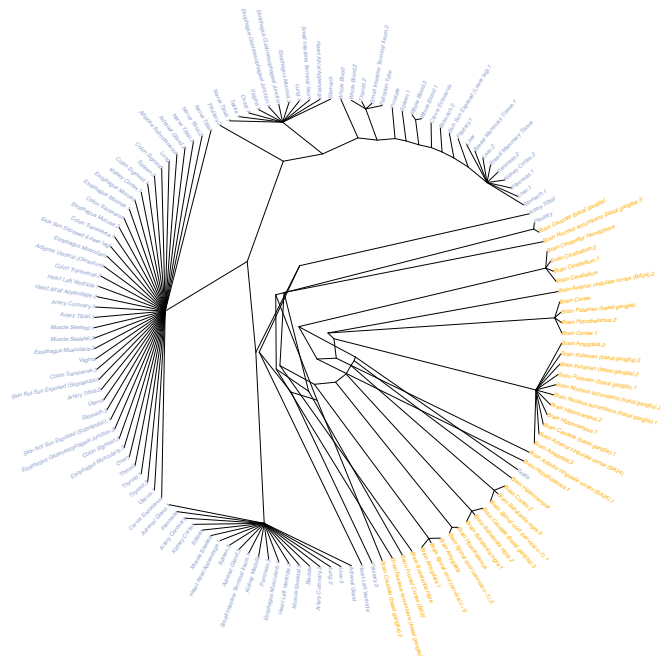

Supplement: Supplementary file 1 [file diagnostics-14-02541-s001.zip › Supplementary Figure 5.pdf]
